# Cholera in Persia

**Published:** 1846-12

**Authors:** 


*Cholera in Persia.*—The last Levant packet which arrived at Malta brought the intelligence that the cholera had broken out in Persia, particularly at Teheran, where one of the brothers of the Schah has fallen. The Schah, with his court, had sought refuge in the mountains. It is feared that, as in 1832, this scourge may again visit Asia Minor and the Mediterranean.

The latest intelligence from Aden announces that the cholera had disappeared from that place, and that the troops were healthy.—*Lond. Med. Gaz.* —*Ibid.*

